# The Protective HIV-1 Envelope gp41 Antigen P1 Acts as a Mucosal Adjuvant Stimulating the Innate Immunity

**DOI:** 10.3389/fimmu.2020.599278

**Published:** 2021-02-03

**Authors:** Lin Xu, Daniela Tudor, Morgane Bomsel

**Affiliations:** ^1^ Laboratory of Mucosal Entry of HIV-1 and Mucosal Immunity, Department of Infection, Immunity and Inflammation, Cochin Institute, CNRS UMR 8104, Paris, France; ^2^ INSERM U1016, Paris, France; ^3^ Université de Paris, Paris, France

**Keywords:** adjuvant, intra-nasal vaccination route, IgA, mucosa, Th2-cytokine thymic stromal lymphopoietin, HIV-1 gp41, P1, microRNA-4485

## Abstract

Mucosal nasal vaccine development, although ideal to protect from pathogens invading mucosally, is limited by the lack of specific adjuvant. We recently used P1, a conserved region of HIV-1 gp41-envelope glycoprotein, as efficient antigen in a prophylactic HIV-1 mucosal vaccine applied nasally. Herein, P1 immunomodulation properties were assessed on human nasal mucosal models by measuring induction of cytokine and chemokine production, intracellular signaling pathways, mucosal dendritic cell (DC) activation, and T cell proliferation. P1 adjuvant properties were evaluated by quantification of antigen-specific B cell responses against a model antigen in an *in vitro immunization model.* We now demonstrated that P1 has additional immunological properties. P1 initiates immune responses by inducing nasal epithelial cells to secrete the Th2-cytokine thymic stromal lymphopoietin (TSLP), a described mucosal adjuvant. Secreted TSLP activates, in turn, intracellular calcium flux and PAR-2-associated NFAT signaling pathway regulated by microRNA-4485. Thereafter, P1 induces mucosal dendritic cell maturation, secretion of TSLP in a TSLP-receptor (R)-dependent autocrine loop, but also IL-6, IL-10, IL-8, CCL20, CCL22, and MMP-9, and proliferation of CD4+ T cells. Finally, P1 acts as an adjuvant to stimulate antigen-specific B cell responses *in vitro.* Overall, P1 is a multi-functional domain with various immuno-modulatory properties. In addition to being a protective vaccine antigen for HIV prevention, P1 acts as adjuvant for other mucosal vaccines able to stimulate humoral and cellular antigen-specific responses.

## Introduction

Although most human pathogens initiate infection at mucosal sites, only a few licensed mucosal vaccines have been established so far (1). Intranasal immunization, by inducing an antigen-specific immunity in both the mucosal and systemic compartments and by being applied in a atraumatic manner following pulverization, is currently considered as an ideal strategy for prevention against pathogens invading mucosa (2–4). Of note, the early side effects attributed to nasal immunization, including facial nerve paralysis, are no longer a concern since they are attributed to the specific ADP-ribosylating toxin-based adjuvant used in these studies rather than to the nasal immunization route (4–6). Mucosal immunization is highly compartmentalized with unique pathways linking the inductive and effector sites (2, 3). In particular, nasal vaccination elicits antigen-specific antibody responses in genital tracts (7) and would be therefore beneficial to prevent transmission of sexually transmitted pathogens. Nevertheless, very limited efforts have been made to understand the mechanisms by which nasal vaccines and dedicated adjuvants activate the local nasal innate and adaptive immunity as a first step to establish an effective vaccination.

P1 is a conserved 35 amino acid peptide covering the Membrane Proximal External Region (MPER) of HIV-1 envelope subunit gp41 (8, 9). The MPER is a major target of broadly neutralizing antibodies and thus obviously a very interesting target for an HIV-1 vaccine. In addition, P1 mediates HIV-1 mucosal transcytosis, a principal mucosal entry pathway for HIV-1, by interacting with Galactosyl Ceramide (GalCer), the mucosal receptor of HIV-1 (9–11). Accordingly, we have recently evaluated the protective efficacy of a gp41-subunit-virosome vaccine at mucosal sites in non-human primates (8). This vaccine that used P1 as antigen linked to virosomes, an adjuvant-free vaccine carrier, was applied twice by the intramuscular route followed by two intranasal applications. In the primate model, full protection after repeated vaginal challenges with simian–human immunodeficiency virus (SHIV) correlated with P1/gp41-specific cervicovaginal antibodies, with IgAs blocking transcytosis and IgGs mediating antibody-dependent cellular cytotoxicity (ADCC). In contrast, in protected animals, serum IgGs totally lacked antiviral activities. Furthermore, in a Phase I clinical trial, we found that P1-virosome vaccination induced mucosal P1-specific antibodies with antiviral activities (12). These results highlighted the critical role of mucosal antibodies as the first line of defense against virus entry.

Thymic stromal lymphopoietin (TSLP) is an IL-7-like cytokine considered as a master regulator of the T helper 2 (Th2) inflammatory responses by priming dendritic cells (DCs), especially mucosal ones (13, 14). We and others recently reported that TSLP is secreted by epithelial cells during HIV-1 mucosal transmission following the interaction of the viral envelope with epithelial cells (15, 16). In turn, TSLP chemo-attract mucosal DCs to the mucosal compartment (16), suggesting that TSLP could modulate the mucosal immune response following mucosal vaccination. Accordingly, in a study using the HIV-1 envelope gp140 as antigen, TSLP acts as a strong mucosal adjuvant in the mouse model (17). TSLP induced a strong humoral response both in serum and at the genital level following intranasal immunization, comparable to the adjuvant effect of cholera toxin (CT) tested in parallel. In addition, a new study reported that all-trans retinoic acid (RA) shows adjuvant activity through TSLP production (18). Furthermore, a recent study showed that TSLP and TSLP-receptor (TSLP-R) were up-regulated in mucosal DCs of mice nasally immunized with pneumococcal surface protein A plus CT (19) and that in TSLP-R knockout mice, the specific IgA response is remarkably reduced. This indicates that TSLP and its receptor are major contributors to the mucosal adjuvant effect of CT and that TSLP- TSLP-R signaling is critical in IgA elicitation.

In the present study, we investigated whether P1, in addition to being an antigen, could act as an adjuvant by first exploring its capacity to stimulate epithelial TSLP production. We then evaluated additional immunomodulatory effects of P1 on human nasal mucosal models, including cytokine and chemokine production, intracellular signaling pathways, mucosal DC activation, T cell proliferation, and antigen-specific B cell responses against a model antigen *in vitro.* Altogether, we report the immunological mechanisms underlying P1-vaccine and the potential of P1 as a nasal mucosal adjuvant.

## Materials and Methods

### Peptides

Peptide P1 (aa 650–685) is derived from the HIV-1 gp41 envelope subunit. P1 clade B (SQNQQEKNEQELLELDKWASLWNWFNITNWLWYIK) is derived from the clade B HXB2 isolate; P1 clade A (SQIQQKKNEQDLLALD KWANLWNWFDISNWLWYIR) from the clade A 99UGA07072 isolate, and P1 clade C (SQTQQEKNEQELLALDSWKNLWNWFSITNWLWYIK) was derived from the clade C Bw96Bw0502 isolate. P1W is a P1 clade B variant with a W666G mutation and P1–5W with all five Ws mutated to G. The scramble peptide sequence comprised the same set of amino acids found in P1 clade B but organized in a random manner (9). Peptides were synthesized with a purity >95% by Biopeptide Co., Inc (San Diego, CA) or United BioSystems (VA, USA).

### Cells

Nasal RPMI 2650 cells (isolated from the human nasal septum, squamous cell carcinoma, ATCC) were grown in MEM*α* (Minimum Essential Medium *α*, Thermo Fisher) supplemented with 10% fetal calf serum (FCS, Eurobio, Courtaboeuf, France) and 1% penicillin/streptomycin.

Primary human nasal epithelial cells (HNECs, purchased from PromoCell, Heidelberg, Germany) were isolated from nasal septum or adenoids of healthy donors. Cells from two independent donors were obtained. HNECs were cultured in airway epithelial cell basal medium (PromoCell) and supplemented with airway epithelial cell growth SupplementMix (PromoCell) and only cells from passages 2 to 6 were used.

Monocyte-derived DCs (DCs) were generated from primary human monocytes obtained from PBMCs (purity >98%) as described (11, 20). In brief, human peripheral blood mononuclear cells (PBMCs) were separated from healthy donors blood (EFS, Paris, France), and monocytes were purified from PBMCs by negative selection according to the manufacturer’s instructions (StemCell Technologies, France). DCs were obtained by incubating monocytes for 7 days in complete medium containing GM-CSF (100 ng/ml) and IL-4 (10 ng/ml).

Autologous CD4+ T cells were purified from PMBCs by negative selection according to the manufacturer’s instructions (StemCell Technologies, France) (purity >95%).

### Quantitative RT-PCR for TSLP

The expression of short and long form TSLP was quantified as described (21, 22). Briefly, total RNA was extracted using Trizol. Five hundred nanograms of RNA was treated with ezDNase Enzyme (Thermo Fisher) to remove genomic DNA and reverse transcribed into cDNA using the kit SuperScript IV VILO Master Mix according to the manufacturer’s instructions (Thermo Fisher). Quantitative PCR was performed using reported primers (21) and the PowerUp SYBR Green Master Mix according to the manufacturer’s instructions (Thermo Fisher). Reactions were performed in triplicates, with glyceraldehyde-3-phosphate dehydrogenase (GAPDH) as the internal control. Amplification, data acquisition, and analysis were carried out using the LightCycler 480 Software (Roche, Mannheim, Germany). The levels of TSLP mRNA were normalized to the levels of GAPDH using the ΔCt method (23) and were presented as 2^−ΔCt^ values.

### MicroRNA Microarray Analysis

Confluent HNECs in 12-well plates were stimulated with medium or P1 (clade B, 125 μM) for 6 h at 37°C. Total RNA was extracted using Trizol. Before analysis, lfTSLP RNA up-regulation was confirmed by qPCR as described above and RNA quality was assessed with Agilent 2100 bioanalyzer according to the manufacturer’s instructions (Agilent Technologies). Three untreated and treated paired samples from three independent experiments were analyzed by GeneChip miRNA 4.0 arrays (Affymetrix, Thermo Fisher) containing probes for 2,578 human mature microRNAs and 2,025 premature microRNAs (https://assets.thermofisher.com/TFS-Assets/LSG/brochures/miRNA_4-0_and_4-1_datasheet.pdf). Potential microRNA targets were analyzed with the Ingenuity Pathway Analysis (IPA) software (Qiagen).

### MiR-4485 Quantification and Knockdown

The quantification and knockdown of microRNA were performed as previously described with some modifications (16). Briefly, total RNA was purified using MinElute PCR Purification Kit (Qiagen), the expression level of miR-4485 was quantified with TaqMan Small RNA Assays (Thermo Fisher). Reactions were performed in triplicates, and U6 was used as endogenous control. In order to knock down miR-4485, 70% confluent HNEC cells were transfected with anti-miR-4485 inhibitor (67 nM, Qiagen) or mock inhibitor (miSCRIPT inhibitor negative control, 67 nM, Qiagen) using Lipofectamine RNAiMAX (Invitrogen) as described by the manufacturer. 36 h after transfection, miR-4485 expression, when quantified as described above, was reduced by 50–60% in anti-miR-4485 transfected cells as compared to anti-miR control (n = 3 independent experiments).

### Signaling Inhibitors

Confluent HNEC cells in 24-well plate were pre-incubated with inhibitors for 1 h at 37°C prior to P1 treatment. Inhibitors, namely dexamethasone (Dex) a NF-*κ*B and MAPK inhibitor (used at 100 nM), and cyclosporin A (CsA) a calcineurin inhibitor (used at 1.5 μM), were from Invivogen and used at the manufacturer’s recommended concentrations. ENMD-1068 (PAR-2 antagonist, Enzo Life Science) was used at 50 μg/ml as described (24, 25).

### Calcium Measurement

70–80% confluent RPMI 2650 or HNEC cells in 24-well plates were loaded with 2 mM Fura-2/AM (Molecular Probes) in basal medium without serum/growth factors for 1 h at 37°C. Cells were washed twice with mammalian saline (26), and measurements were performed in complete medium supplied with HEPES (10 mM) and CaCl_2_ (2 mM) as described (26). Images were acquired with an inverted fluorescence microscope (Observer Z1, Zeiss, Germany) and analyzed with MetaMorph software (27). Calcium was measured every 5 s by video fluorescence imaging. Results were expressed as 340 nm to 380 nm fluorescence ratio and normalized to the baseline, *i.e.* ratio at time zero was set as 1.

### Cytokines and Chemokine Quantification

TSLP, IL-25/IL-17E, IL-33, IFN-*γ*, IL-10, IL-12/23p40, IL-4, IL-5, IL-6, IL-13, TNF-α, MMP-9, IL-8/CXCL8, MIP-3*α*/CCL2, MCP-1/CCL20, MDC/CCL22, TARC/CCL17, APRIL, and BAFF were measured in culture supernatants from the indicated experiments with custom multiplex Luminex assays (Bio-techne) according to the manufacturer’s instructions. Additionally, the indicated TSLP was measured in culture supernatants by enzyme-linked immunosorbent assay (ELISA) with a limit of detection of 8 pg/ml (Thermo Fisher) according to the manufacturer’s instructions.

### DC–EC Co-Culture and DC Activation

Three DC culture systems were developed. Monocytes derived DCs (5 × 10^5^ cells) were incubated for 24 h in medium alone and considered as non-mucosal DCs (DCs) or co-cultured with nasal epithelial cell (RPMI-2650 cell line) monolayer in 24-well plate (DC–EC or eduDC systems for 24 h at 37°C. In turn, DCs were either further cultured with ECs during P1 stimulation (DC–ECs) or separated from EC and transferred into a new plate (eduDCs) for further P1 treatment. Subsequently, P1 (clade B, 125 μM) or medium were added to each of the DCs, DC–ECs or eduDCs cultures for 16 h. DCs were collected for surface staining with allophycocyanin (APC)-conjugated anti-CD86, R-phycoerythrin (PE)-conjugated anti-CD83, APC-conjugated anti-TSLPR, PE-conjugated anti-IL-7R*α* antibodies (all from Bio-Techne). Specific labeling was quantified by flow cytometry using a Guava EasyCyte flow cytometer and the InCyte software (Merck) described (28). Culture supernatants were collected and frozen at −80°C for subsequent cytokine and chemokine analyses.

### DC-T Cell Co-Cultures

DCs and confluent ECs were co-cultured overnight as described above, and DC–EC or eduDC was further incubated with P1 (clade B, 125 μM) or medium for 24 h. Then, DCs were separated and incubated with autologous CD4+ T cells pre-labeled with CFSE (Thermo Fisher) according to the manufacturer’s instructions, at a ratio of 1:5 (DC/T). After 5 days of culture, CD4+ T cell proliferation was analyzed by flow cytometry as described (29, 30) using Phytohaemagglutinin (PHA) (5 μg/ml) as positive control.

### *In Vitro* Immunization Assay

*In vitro* immunization assay was performed as reported (31) with modifications. Briefly, 1 × 10^6^ CD8-depleted PBMCs (Human CD8 Depletion Cocktail, StemCell Technologies, France) were co-cultured for 24 h with RPMI 2650 cells (1 × 10^5^) pre-seeded in 48**-**well plates for 48 h. Then, ovalbumin (OVA, EndoFit Ovalbumin, 10 μg/ml, Invivogen) alone, OVA together with P1 (5 μM, 25 μM, 125 μM), OVA together with P1 mutant (P1mut, 125 μM), or medium were added to in RPMI 1640 medium supplemented with Non-Essential Amino Acids (NEAA solution, Thermo Fisher), IL-4 (10 ng/mL), IL-2 (10 UI/mL) and 2-mercaptoethanol (20 μM) for 7 days.

For the detection of OVA-specific B cells, at the time points indicated, PMBCs were surface stained with ovalbumin conjugated to fluorescein (OVA-FITC, 20 ug/ml, Thermo), PE-conjugated mouse anti-human CD20 (BD Biosciences, CA, USA), APC-conjugated goat anti-human IgA or donkey anti-human IgG (Jackson ImmunoResearch, PA, USA) as indicated by the manufacturer. Specific labeling was quantified by flow cytometry with a Guava EasyCyte flow cytometer (Merck-Millipore), and analyzed with the dedicated InCyte software, using the following strategy: CD20+ B cells were first gated and cells double positive for OVA-FITC+ and APC-conjugated anti-IgA or anti-IgG were determined as OVA-IgA or IgG-specific B-cells, respectively.

### Statistical Analysis

Data are presented as mean ± SEM of at least three independent experiments. Statistical significance was analyzed by the two-tailed Student’s t-test with the GraphPad Prism software.

## Results

### P1 Induces TSLP Secretion in Nasal Epithelial Cells by Interacting With Galactosyl Ceramide

**Figure 1:**
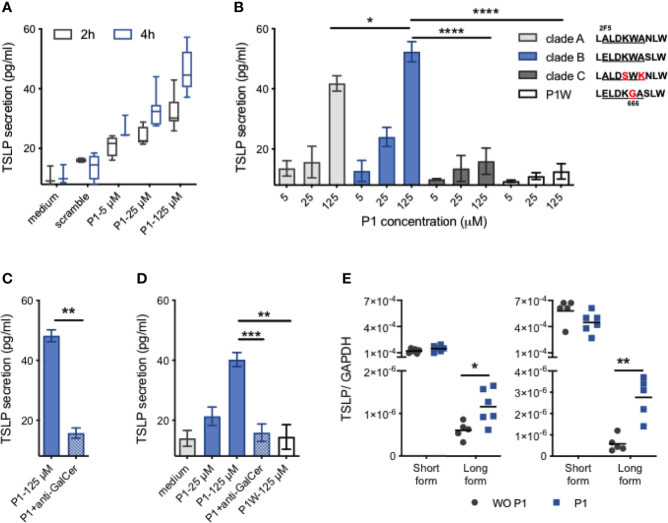
P1 induces TSLP expression in nasal epithelial cells. **(A)** Confluent RPMI 2650 cells were cultured with P1 peptide (5 μM, 25 μM, 125 μM) or scramble peptide (125 μM), for 2 h or 4 h. TSLP secretion in culture supernatants was quantified by ELISA. Data are presented as box-and-whisker plots. **(B)** RPMI nasal cells were cultured with HIV-1 clade **(A–C)** derived P1 peptides or the mutated P1–5W peptide at increasing concentrations for 4 h. Inset: P1 key amino acids (661–670) corresponding to the broadly neutralizing 2F5 and 4E10 IgG epitopes with clade-specific mutations are aligned. **(C)** RPMI nasal cells were pre-incubated with anti-galactosyl ceramide (GalCer) antibody for 30 min at 37°C before stimulation with each P1 peptide. **(D)** Primary HNEC cells were cultured with P1 peptide (5 μM, 25 μM, 125 μM), with or without anti-GalCer pre-incubation, or P1W peptide (125 μM). **(E)** Relative mRNA expression of the short form (sf) and long form (lf) TSLP in RPMI nasal cells (left) and HNEC cells (right). Cells were stimulated with (P1) or without (WO P1) P1 for 4 h, 37°C. Data in **(B–D)** are presented as mean ± SEM (n = 3–8 independent experiments. paired student’s t-test *p < 0.05, **p < 0.01, ***p < 0.001, ****p < 0.001).

We first investigate whether P1 induced TSLP secretion in nasal epithelium. Therefore, we cultured human nasal epithelial cells (RPMI 2650) with P1 clade B for 2–24 h at 37°C and analyzed the culture supernatants for TSLP secretion. Compared with the medium and scramble peptides used as negative controls, P1 up-regulates TSLP secretion in a dose-dependent manner from 2 to 4 h (**Figure 1A**). At 125 μM, when P1 adopts a trimeric oligomerization state (9), P1 induces a significantly higher secretion of TSLP than in a monomeric state (at 5 μM and 25 μM). TSLP secretion occurs rapidly within hours post stimulation reaching a plateau from 4 to 24 h.

Although the P1 sequence is relatively conserved, in contrast to highly mutated regions of HIV-1 envelope gp120, the P1 sequence varies between HIV-1 clade A that is common in West Africa, clade B that predominates in Europe and the USA, and clade C that predominates in Africa and China (**Figure 1B**). Consequently, we next analyzed whether TSLP secretion was restricted to clade B derived P1 or would also be stimulated by P1 derived from clade A and C viruses (**Figure 1B**). Secretion of TSLP induced by clade A compared to clade B P1 is reduced by 20% (41.8 ± 2.6 pg/ml for clade A, 52.3 ± 3.4 pg/ml for clade B P1 at 125 µM, *p* < 0.05, n = 5) whereas P1 clade C failed to induce TSLP secretion. P1 clade C differs from P1 clades B and A by the ELDKW motif, we have previously shown to be determinant in P1 clade B binding to galactosyl ceramide (GalCer), the epithelial HIV-1 receptor (9, 10). We thus hypothesized that P1 clades B and A interaction with GalCer initiated TSLP secretion. Accordingly, P1 clade B mutated in W666G (P1W) that fails to interact with GalCer (9) completely loses the capacity to induce TSLP secretion. Furthermore, when the interaction between P1 and GalCer was blocked by pre-incubation with anti-GalCer antibody, TSLP production is entirely blocked, confirming that TSLP secretion is initiated by P1 interaction with GalCer (**Figure 1C**). Importantly, P1 stimulation also induces primary human nasal epithelial cells (HNECs) to secrete TSLP in a GalCer-dependent manner (**Figure 1D**).

### Long-Form TSLP Is Up-Regulated After P1 Stimulation

Two transcript variants of TSLP, namely the short (sfTSLP) and the long (lfTSLP) forms, were recently identified (32). The expression of sfTSLP has been suggested to be constitutive and homeostatic, whereas the lfTSLP leads to proinflammatory responses (32). We thus investigated which form(s) of TSLP was up-regulated by P1 stimulation of nasal epithelial cells. When analyzed at the transcriptional level in nasal RPMI and primary HNEC cells, the expression of sfTSLP and lfTSLP differs by a factor >10^2^. Upon P1 stimulation of both nasal RPMI cells and primary HNECs, the level of the sfTSLP transcript remains unchanged (**Figure 1E**). In contrast upon P1 stimulation, the level of lfTSLP transcription in both nasal RPMI cells and HNECs increased by 1.9 (p = 0.02, n = 5) and 5.9-fold (p = 0.004, n = 6), respectively, compared to unstimulated cells. Altogether, these results indicate that P1 up-regulates lfTSLP selectively at a transcription level.

### P1-Induced lfTSLP Expression Is Regulated by miR-4485, Calcineurin, and PAR-2

Next, we investigated the intracellular mechanisms leading to TSLP expression after P1 interaction with GalCer. We concentrated on primary HNECs as its increase in lfTSLP transcription level upon P1 stimulation is higher compared to that in nasal RPMI cells (**Figure 1E**).

**Figure 2:**
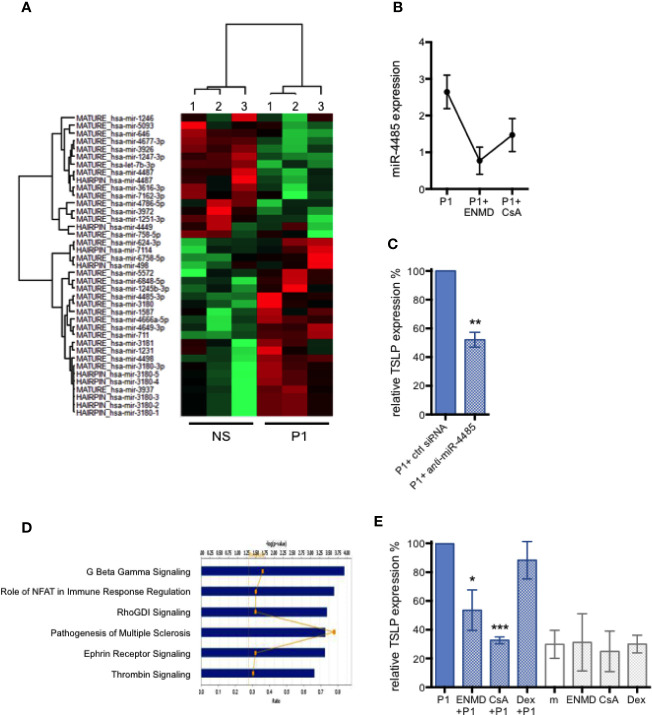
MicroRNA-regulated P1-induced TSLP expression and corresponding signaling pathways in nasal epithelial cells. **(A)** Heatmap showing 39 differentially expressed miRNAs in P1 stimulated (P1) HNEC cells compared with control unstimulated cells (NS). Only differentially expressed microRNAs with *p* value <0.05 from paired student’s t-test, and a cut-off of fold change >1.3 are included. n = 3 independent experiments with HNECs from two different donors. **(B)** PAR-2 antagonist ENMD-1068 (ENMD) and calcineurin inhibitor cyclosporine A (CsA) decrease the expression of P1-induced miR-4485 as measured by qPCR. **(C)** miR-4485 knock-down in HNECs by siRNA transfection inhibited P1-induced TSLP expression, measured by qPCR, by about 50%. **(D)** Top six target pathways predicted by Ingenuity Pathway Analysis sorted by their *p* values. The ratio presented is defined as the number of the differentially miRNAs found in the P1-treated cells over the total number of miRNA involved in each of the pathway. **(E)** P1-induced TSLP expression, measured by qPCR, is significantly reduced in the presence of CsA, an inhibitor of NFAT and ENMD, an antagonist of PAR-2, but not by Dexamethasone (Dex), an inhibitor of NF-κB and MAPK. m: medium. Data are presented as mean ± SEM n ≥ 3 independent experiments paired student’s t-test *p < 0.05, ** *p* < 0.01, ***p < 0.001).

We have previously shown that the non-coding microRNA miR-375 controls TSLP expression in primary human foreskin keratinocytes (16), as it does in human intestinal cell lines (33). When tested in primary HNECs, we found that the TSLP secretion induced by P1 described above is not accompanied by a change in miR-375 expression. We thus further investigated the microRNA profiles upon nasal epithelial HNEC stimulation by P1 after treatment with or in the absence of P1 for 6 h, comparatively by microRNA array analysis. As a result, 39 microRNAs are differentially expressed with a fold change ranging from 1.3 to 9.15 when p <0.05, including 23 up-regulated and 16 down-regulated genes (**Figure 2A**, **Table 1**).

**Table 1:** MicroRNAs induced (left) or repressed (right) in HNECs treated with P1.

gene ID	fold (P1/n.s.)**	p value ***	gene ID	fold (P1/n.s.)	p value
hsa-mir-4485-3p	9.15	0.049	hsa-mir-3972	−1.95	0.020
hsa-mir-5572	1.95	0.030	hsa-mir-4487	−1.77	0.013
hsa-mir-3180	1.67	0.030	hsa-mir-4786-5p	−1.62	0.024
hsa-mir-3937	1.65	0.035	hsa-mir-1246	−1.56	0.012
hsa-mir-1587	1.56	0.002	hsa-mir-5093	−1.53	0.026
hsa-mir-6758-5p	1.56	0.012	hsa-mir-7162-3p	−1.52	0.018
hsa-mir-711	1.54	0.001	hsa-let-7b-3p	−1.48	0.030
hsa-mir-3180-3p	1.54	0.023	hsa-mir-3926	−1.47	0.003
hsa-mir-3180-5*	1.47	0.011	hsa-mir-758-5p	−1.40	0.011
hsa-mir-3180-4*	1.46	0.021	hsa-mir-4677-3p	−1.40	0.004
hsa-mir-3180-1*	1.44	0.039	hsa-mir-3616-3p	−1.40	0.003
hsa-mir-3180-2*	1.44	0.039	hsa-mir-1247-3p	−1.36	0.037
hsa-mir-3180-3*	1.44	0.039	hsa-mir-646	−1.35	0.016
hsa-mir-4666a-5p	1.41	0.040	hsa-mir-4449*	−1.33	0.023
hsa-mir-7114*	1.38	0.041	hsa-mir-4487*	−1.32	0.046
hsa-mir-3181	1.37	0.042	hsa-mir-1251-3p	−1.31	0.010
hsa-mir-6848-5p	1.35	0.026			
hsa-mir-1231	1.34	0.013			
hsa-mir-4649-3p	1.33	0.035			
hsa-mir-4498	1.33	0.036			
hsa-mir-624-3p	1.31	0.026			
hsa-mir-1245b-3p	1.31	0.045			
hsa-mir-498*	1.31	0.007			

Hairpin format pre-mature microRNA; **n.s., non stimulated; ***p values were calculated from paired t-test.

Remarkably, in the microRNA array analysis, the highest up-regulated gene upon P1 stimulation is the miR-4485-3p with a n> nine-fold increase (**Table 1**). We validated this up-regulation by qPCR resulting in an increase in miR-4485-3p expression by 2.6 ± 0.8-fold (n = 4) upon P1 stimulation (**Figure 2B**). MiR-4485-3p is a relatively newly described microRNA that is poorly characterized at the experimental level. The only described activity of miR-4485-3p is to regulate mitochondrial functions, suggesting a role in tumor suppression (34). Thus, we first evaluated whether this microRNA controlled TSLP expression. Therefore, primary HNECs were transfected with a specific siRNA to inhibit miR-4485-3p expression before P1 stimulation. As a result, knocking down miR-4485-3p by 50–60% decreases in turn P1-induced TSLP expression by 48 ± 10% (p < 0.01, n = 4), compared to cells transfected with a mock inhibitor (**Figure 2C**).

Bioinformatic analyses were conducted to further elucidate the mechanisms by which P1 modulates all identified microRNAs and subsequent intracellular signaling pathways. The genes predicted to be targeted by identified microRNAs participate in several signaling pathways, the five principals including G protein-coupled receptor (GPCR)**-**associated signaling, Nuclear factor of activated T-cells (NFAT) signaling, Rho GDP signaling, Ephrin receptor signaling, and thrombin signaling (**Figure 2D**).

Corroborating this predictive analysis designating NFAT, GPCR, and thrombin (PAR associated) pathways (35) as the main ones induced by P1 stimulation, it has been described that in keratinocytes, TSLP production is regulated by Ca^2+^-dependent NFAT signaling itself triggered by the activation of GPCR protease-activated receptor 2 (PAR-2) (36). Thus, we next evaluated experimentally whether inhibitors specific to these pathways also reduced P1-induced TSLP expression in primary HNECs. Therefore, HNECs were pre-treated with the calcineurin inhibitor Cyclosporine A (CsA) or with the PAR-2 antagonist ENMD-1068 prior to P1 stimulation. Accordingly, TSLP expression was reduced by 67 ± 4% (*p* < 0.001, n = 3) upon CsA pre-treatment and by 46±14% (*p* < 0.05, n = 3) following ENMD-1068 pre-treatment (**Figure 2E**). Furthermore, CsA and ENMD-1068 inhibitors also blocked the up-regulation of miR-4485-3p (**Figure 2B**). In contrast, blocking NF-*κ*B and MAPK with Dexamethasone (Dex) had no effect on TSLP expression (**Figure 2E**). These results provide direct and indirect evidence that miR-4485-3p, calcineurin, and PAR-2**-**mediated signaling tightly correlate with P1-induced TSLP expression.

**Figure 3:**
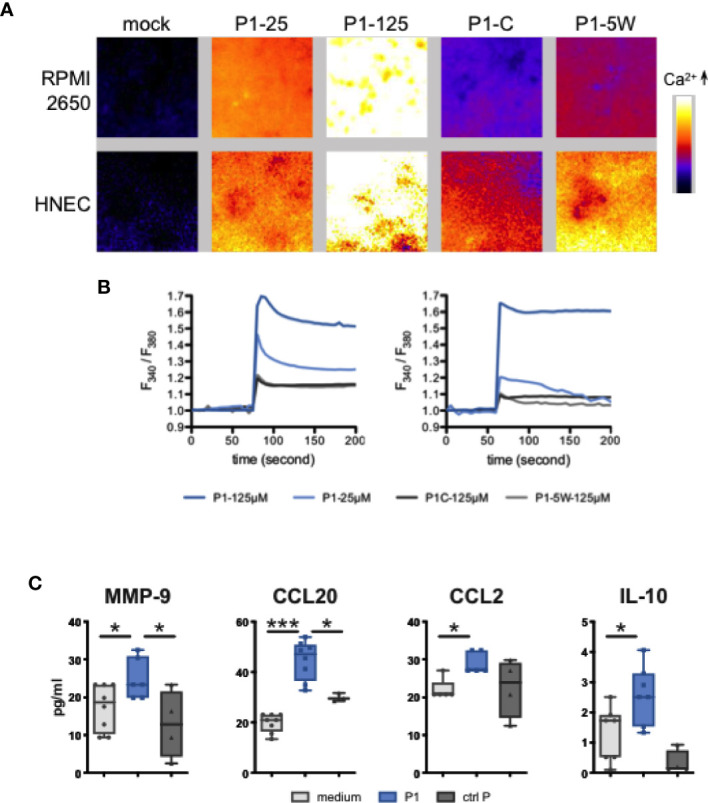
P1 induces calcium flux and cytokine/chemokine secretion in nasal epithelial cells. **(A, B)** Calcium fluxes in response to P1 stimulation**. (A)** Representative images of Fura-2/AM loaded epithelial cells treated with medium (mock), P1 (5 μM, 25 μM, 125 μM), P1 of clade C (P1-C, 125 μM), and mutated P1 (P1–5W, 125 μM). **(B)** Calcium was measured every 5 s by video fluorescence imaging and displayed as the ratio of 340 and 380 nm excitation signals. Representative trace out of n = 3 independent experiments of calcium fluxes in Fura-2/AM loaded epithelial cells (left RPMI, right HNEC cells) treated with P1 and other indicated peptides. **(C)** Cytokine and chemokine release from nasal epithelial cells upon P1 stimulation. P1 elicits MMP-9, CCL20, CCL2, IL-10 secretion in nasal epithelial cells. Cells were incubated with medium, P1 (clade B, 125 μM) or control peptide: scramble P1, P1–5W or P1 clade C, 125 μM (ctrl P) for 24 h. MMP-9, CCL20, CCL2, IL-10 secretions were measured by multiplex Luminex assay. Data are presented as mean ± SEM **(A, B)** Data are presented as box-and-whisker plots from n = 5 independent experiments; paired student’s t-test *p < 0.05, ***p < 0.001.

To further confirm that P1 activates calcineurin, we investigated whether, in nasal epithelial cells, P1 induces calcium fluxes that generally cause calcineurin activation (37). Accordingly, using fluorescent dye Fura-2/AM imaging technology, we observed in both nasal RPMI cells and primary HNEC cells that P1 treatment induces an immediate extracellular calcium influx in a concentration-dependent manner (125 μM *vs* 25 μM of P1, n = 3) (**Figures 3A, B**). In contrast, treatment with control peptides (P1–5W mutant and P1 clade C, both at 125 μM) fails to raise the calcium level significantly.

Together, these data indicated that in nasal epithelial cells, P1-stimulated TSLP expression is regulated by miR-4485 *via* a Ca^2+^-dependent NFAT signaling pathway through the interaction with PAR-2 receptor.

### P1 Further Stimulates Epithelial Secretion of MMP-9, CCL20, CCL2, and IL-10

We next investigated whether, in addition to TSLP, P1 could stimulate epithelial secretion of additional immune factors prone to attract antigen presenting cells (APCs). Therefore, nasal RPMI cells where incubated with P1 (125 μM) and after 24 h, the cell culture medium was analyzed for interleukin (IL)-25/IL-17E, IL-33, IFN-*γ*, IL-10, IL-12/23p40, IL-4, IL-5, IL-6, IL-13, TNF-α, Matrix metalloproteinase 9 ((MMP-9), IL-8/CXCL8, MIP-3*α*/CCL2, MCP-1/CCL20, MDC/CCL22, TARC/CCL17, APRIL, and BAFF by Luminex technology. As a result, P1 selectively induced the secretion of MMP-9, CCL20, CCL2, and IL-10 (**Figure 3C**). Furthermore, as observed for P1-induced TSLP secretion, P1W and P1 clade C were unable to stimulate significant MMP-9, CCL-20 CCL2, or IL-10 production. Together with TSLP (16), this set of immune factors could facilitate recruitment of APCs to the mucosal surface for initiation of an immune response, since CCL20 and CCL2 chemo-attract macrophages and immature DCs and MMP-9 degrade the extracellular matrix and facilitate the migration of immune cells in or out the epithelium. Treg cells have IgA-inducing functions and require RA, TGF-b1, IL-10, and TSLP from the intestinal epithelial cells and DCs. So, we assumed IL-10 released from either EC or DC may contribute to IgA class switching (Gutzeit, Magri, et al., 2014).

### P1 Activates Human Dendritic Cells in a Nasal Mucosal Model

APCs link the innate and adaptive immune systems and determine the polarization of the immune responses. APCs are thus a key target in vaccine and adjuvant development (38). DCs being the most abundant APCs in airway mucosa (39), we further investigated mucosal DC responses to P1.

**Figure 4:**
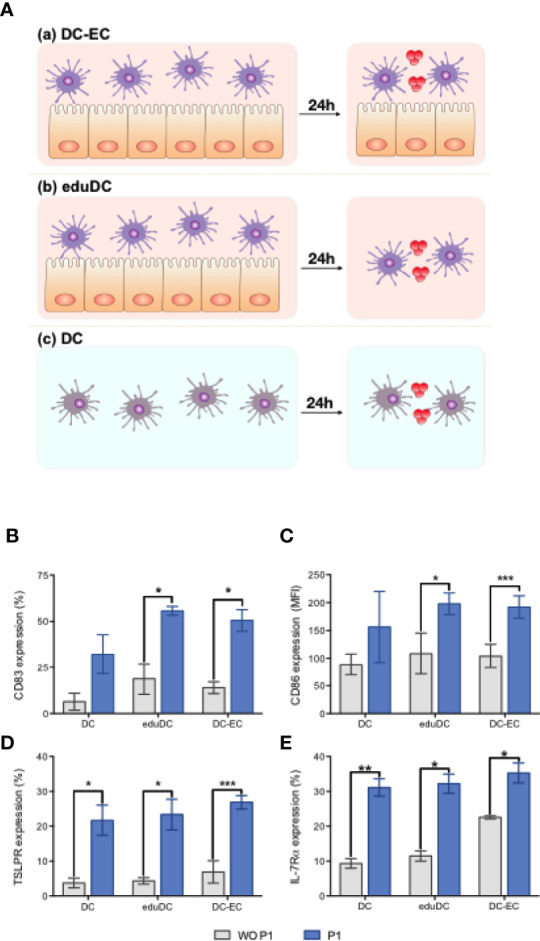
P1 induces mucosal dendritic cell (DC) maturation. **(A)** Experimental models of mucosal DC activation by P1. Experimental model evaluating (a) mucosal DC generated by continuous contact between epithelial (EC) and DC (DC–EC): DCs, co-cultured with EC monolayer (RPMI-2650 cell line) for 24 h, were treated with P1 or medium for an additional 16 h; (b) mucosal DC generated by an initial education by EC following a time-limited contact between EC and DC (eduDC): after co-cultured with ECs for 24 h as above, DCs were separated from EC and subsequently treated with P1 or medium for an additional 16 h. (c) non-mucosal DC (DC): DCs were cultured alone with medium for 24 h prior to stimulation by P1 or medium for 16 h. **(B–E)** P1 activates DCs by up-regulating the expression of surface markers. Expression of CD83 **(B)**, CD86 **(C)**, TSLPR **(D)**, IL-7Ra **(E)** on DCs obtained after culture within the three models described in **(A)** and quantified by flow cytometry with (blue bars) or without (WO) (gray bars) P1 stimulation. CD86 expression is shown as mean fluorescence intensity (MFI). Data are presented as mean ± SEM (n > 3 independent experiments; paired student’s t-test *p < 0.05, **p < 0.01, ***p < 0.001).

It has been suggested that mucosal DCs display unique functions due to the local microenvironment, especially at mucosal level (40). In particular, mucosal DCs modulate their functions by interacting with epithelial cells (ECs) including *via* epithelial secretion of TSLP (33, 41). Thus, we established a simplified mucosal DC model, by co-culturing DCs and nasal ECs (RPMI-2650 cell line), thereby mimicking the nasal mucosal environment as depicted in **Figure 4A**. DCs were first ‘educated’ by a 24 h co-culture with ECs. Subsequently, these ‘educated’ DCs were either maintained in culture with ECs and referred to as DC–EC, or separated from the epithelium and referred to as eduDC. Alternatively, DCs only cultured with medium represented ‘non-mucosal’ DCs.

Each type of DCs was stimulated with P1 overnight, and the expression of maturation markers was assessed by flow cytometry. Compared to untreated cells, P1-treated mucosal DCs, either DC–EC or eduDCs, show a significant up-regulation of co-stimulatory molecules CD83 (**Figure 4B**) and CD86 (**Figure 4C**). In contrast, P1 has no effect on ‘non-mucosal’ DCs. Surprisingly, P1 also significantly enhanced the expression of TSLP receptor, with both chain TSLP-R (**Figure 4D**) and IL-7R*α* (**Figure 4E**) being up-regulated on the DCs in all three models.

**Figure 5:**
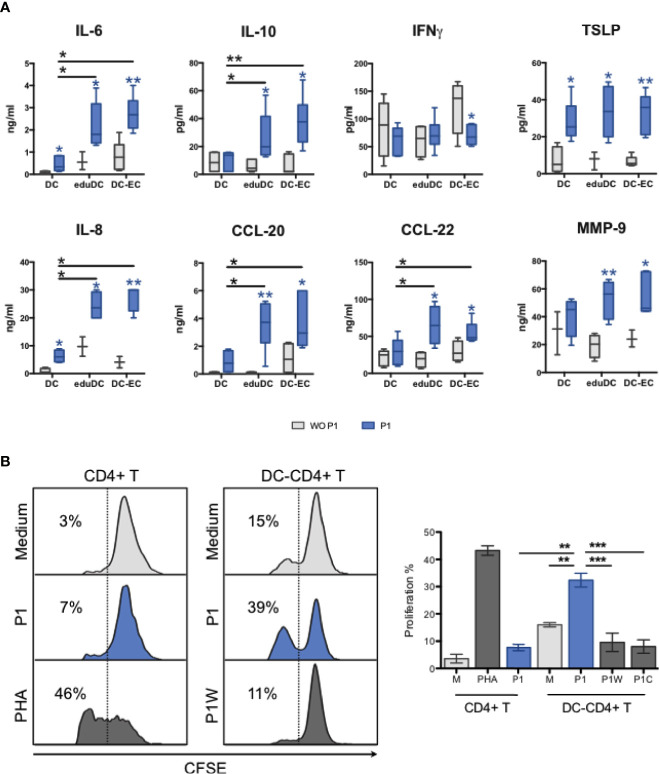
P1 stimulation of mucosal DCs results in CD4+T cell proliferation. **(A)** Cytokine and chemokine production of P1-treated DC models. IL-6, IL-10, IFNg, TSLP, IL-8, CCL-20, CCL-22, and MMP9 were quantified by a multiplex Luminex assay. The blue star (*) indicates significant differences (*p* values <0.05, student’s t-test) in each model between P1 (blue bars) and mock (gray bars) treatments, whereas the black star (*) compares P1**-**treated cells in different models. Data are presented as box-and-whisker plots from n > 3 independent experiments, *p* values <0.05, paired student’s t-test). **(B)** P1-treated mucosal DCs stimulate the proliferation of CD4+ T cells. CD4+ T cell proliferation was determined by flow cytometry according to CFSE intracellular concentration. Data are represented as proliferating CD4+ T cells as % of total CD4+ T cells. Phytohaemagglutinin (PHA) was used as positive control. Data are presented as mean ± SEM (n > 3 independent experiments; paired student’s t-test *p < 0.05, **p < 0.01, ***p < 0.001).

The cytokine and chemokine secretion profiles were also studied in these models, comparatively. Compared with non-mucosal DCs, P1 induces a significant increase in IL-6, IL-8, IL-10, CCL20, CCL22, and MMP-9 secretion in eduDC and DC–EC models as well as that of TSLP secretion, although more modest. In contrast, IFN-*γ* secretion remains unchanged upon P1-stimulation or slightly decreases in DC–EC (**Figure 5A**). In addition, several cytokines, such as IL-25, IL-33, IL-4, IL-5, remain undetectable whatever the model, whereas others, such as IL-12, IL-13, CCL2, CCL17, TNF-α, APRIL, and BAFF, are secreted equally in all three models.

Activated DCs are known to stimulate T-cell proliferation to initiate an adaptive immune response, both *in vivo* and *in vitro* (15, 29, 30). Therefore, we further assessed if P1 activated mucosal DCs could promote T cell proliferation. As a result, P1 primed eduDCs induced the proliferation of autologous CD4+ T cells, whereas treatment with control peptides (P1W mutant and P1 clade C) or P1 stimulation on CD4+ T cells alone has no effect (**Figure 5B**). Similar results were observed with DC–ECs, in agreement with the similar cytokine profiles between DC–EC and eduDC as described above.

Altogether, these results show that P1 activates mucosal DCs specifically, resulting in Th2 cytokine and chemokine secretion, and in CD4+ T cell proliferation.

### P1 Acts as an Adjuvant to Stimulate Antigen-Specific Humoral Responses *In Vitro*

Finally, given that P1 induces various immunomodulatory effects in mucosal cells involved in vaccination at the nasal site, as described above, we investigated whether P1 was able to act as an adjuvant. Using an *in vitro* immunization model with human PBMCs, the capacity of P1 to trigger a humoral immune response against a well-characterized antigen, namely ovalbumin (OVA), was evaluated.

**Figure 6:**
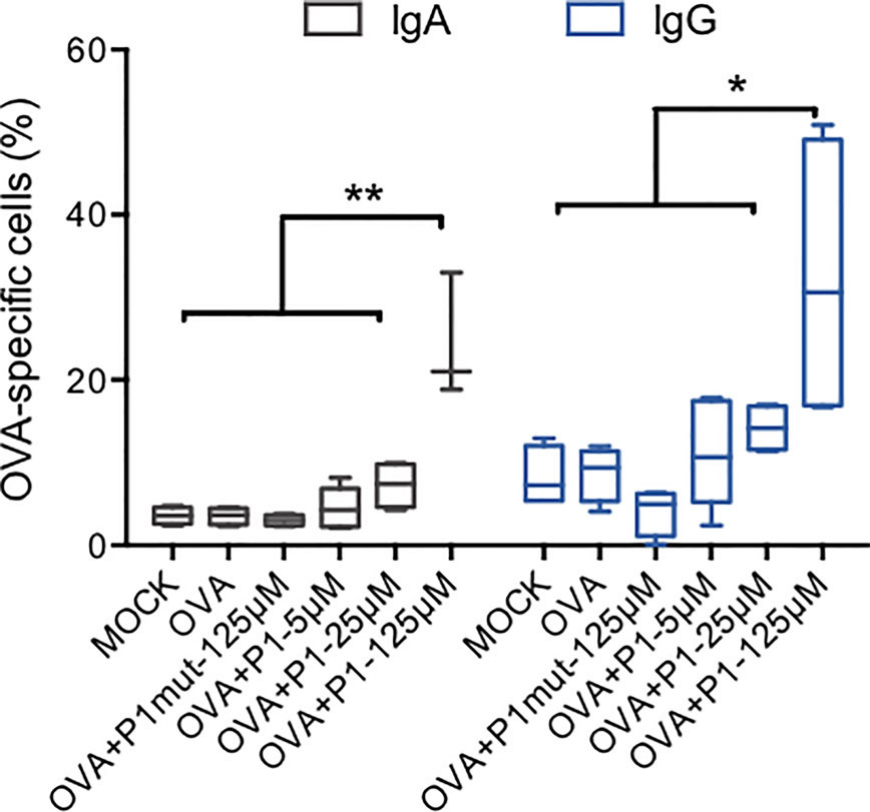
*In vitro* immunization with ovalbumin adjuvanted by P1 peptide. CD8-depleted PBMCs were co-cultured with RPMI nasal cell monolayer for 24 h prior to addition of OVA as an antigen, adjuvanted by P1 at three concentrations or in the presence of mutated P1 (P1mut), in the absence of antigen or adjuvant as negative controls. CD20+ B cells expressing OVA-specific IgA or IgG were quantified by flow cytometry using anti-CD20-PE, FITC-conjugated OVA, and APC**-**conjugated anti-human-IgA or anti-human-IgG. Data are presented as box-and-whisker plots from n = 5 independent donors; paired student’s t-test *p < 0.05, **p < 0.01.

*In vitro* immunization assays have been used to produce specific monoclonal antibodies using a defined antigen complemented with an adjuvant (42). Here, we establish a mucosal immunization model adapted from (29, 31, 43) and using mucosal DCs based on our results presented above. OVA were selected as the model antigen. Therefore, human CD8-depleted PBMCs (n = 5 independent donors) were co-cultured with RPMI 2650 cells for one day to educate DCs, and prior to addition of either medium, OVA, OVA plus P1 mutant (P1mut, 125μM) or OVA plus P1 (5 μM, 25 μM, 125 μM) for seven more days. OVA-specific B cells were quantified by flow cytometry using FITC-conjugated OVA and anti-CD20-PE to gate on OVA-specific B cells. The Ig isotype of surface B cell receptor (BCR) was next characterized by APC-conjugated anti-human-IgA or anti-human-IgG. As shown in **Figure 6**, OVA alone, similarly to medium, failed to induce OVA-specific specific B cells, whereas in the presence of P1, OVA-specific B cells were detected. At 5 μM and 25 μM, the concentration at which P1 remains in the monomeric state, induction of OVA-specific B cells is very limited, whereas, at 125 μM, P1 significantly enhances OVA recognition by B cells due to surface expression of OVA-specific IgA and IgG isotypes. Similar results were obtained when B cells were stained with anti-CD19-PE. Importantly, in the absence of nasal epithelial cells during the *in vitro* immunization, P1 is not able to induce OVA-specific B cells. Within the culture supernatants of day 7, OVA-specific antibody secretion could not be detected by ELISA, most likely because blasts were not formed at this early time point of the immunization and in agreement with the detection of OVA-specific IgA and IgG at the B cell surface, prior to blast differentiation. Hence, P1 appears to act as an adjuvant by promoting the expression of antigen-specific BCR on B cells, which may need additional signals to develop into plasma cells.

## Discussion

In contrast to intensively used bacterial adjuvants, the beneficial immune properties of viral components have been mostly overlooked. Although viruses are commonly considered to trigger PRRs (Pattern recognition receptors) by their genetic materials, viral envelope proteins have also been reported to initiate local inflammation *via* the interaction with TLR-2 and TLR-4 (44–47). These motifs are critical for virus transmission but might also be used to improve vaccine uptake and efficacy.

Here, we reported for the first time about the adjuvant activity of a viral membrane protein and explored the corresponding immunological mechanisms. Therefore, we have characterized the immuno-modulatory properties of P1, a conserved peptide from the HIV-1 glycoprotein gp41 we have previously shown to be a powerful vaccine antigen providing full protection against mucosal HIV infection following intra-nasal immunization (8) and determined its stimulatory activities in human nasal epithelial cells and dendritic cells, the two major targets of nasal vaccination.

Nasal epithelial cells are the first cells encountered by the vaccine applied nasally and are recognized to influence the initiation, regulation, and maintenance of mucosal innate and adaptive immune responses *via* epithelial-derived factors, such as TSLP (33, 41). The P1-induced TSLP secretion by nasal epithelial cell shown here could thus constitute the initial adjuvant activity achieved by P1 used in a nasal vaccine formulation. TSLP offers several advantages for stimulation of the local immunity and induction of specific antibody production, two main goals of mucosal vaccination (18). In particular, TSLP affects polarization towards Th2 immune responses, promotion of IgA class switching, and mediates the generation and maintenance of memory T cells (48). Hence, the introduction of TSLP in a vaccine formulation applied mucosally strongly stimulates the IgA response (19), and TSLP has demonstrated adjuvant activity in a recent HIV-1 vaccine study with intra-nasal administration in the mouse model (17). Recent studies by us and others demonstrated that TSLP is secreted during the initial steps of HIV-1 entry in the genital mucosa following gp120-dependent activation of the TLR-4 pathway through NF-*κ*B signaling (15, 16). Based on the considerable efficacy of our gp41-subunit vaccine comprising virosome, an adjuvant-free vaccine carrier, coupled to gp41 subunits used as antigen, and administered nasally, we decided to investigate whether our vaccine antigen, the gp41-subunit P1, could also stimulate TSLP and thus acts as a mucosal adjuvant.

However, nasal epithelial cells differ from genital ones and thus respond differently to pathogens they are associated with. Hence, nasal epithelial cells cannot be activated by LPS *via* TLR-4 (49). Accordingly, our study revealed that P1 stimulates TSLP production in nasal epithelial cells by interacting rather with GalCer, the HIV mucosal receptor expressed on epithelial cells and dendritic cells (9–11).

We further studied the in-depth mechanism stimulated by P1 interaction with GalCer to identify the cellular regulator of TSLP expression in nasal epithelium. As TSLP secretion by various stimuli is regulated by miRNA (16, 33), we first studied the microRNA transcriptome selectively induced in nasal epithelial cells upon P1 stimulation. We identified miR-4485 as the main miRNA that is differentially increased in nasal cells upon P1 stimulation. Accordingly, blocking miR-4485 abolished P1-induced TSLP secretion. Further prediction-based systematic analysis based on targets of identified miRNAs computed that the PAR-2 associated NFAT pathway was modulated. PAR-2 plays a key role in TSLP release, particularly the PAR-2/ORAI1/NFAT calcium pathway that has been reported to regulate TSLP production in keratinocytes (36). Here, we experimentally established the capacity of P1 to stimulate the PAR-2/ORAI1/NFAT calcium pathway in nasal epithelial cells. Hence, P1-induced TSLP secretion is blocked by PAR-2 and calcineurin specific inhibitors, and P1 induces an intracellular calcium influx. Furthermore, in addition to TSLP, P1 stimulates the specific secretion of CCL20, CCL2, IL-10, and MMP-9 in nasal epithelial cells. In line with these results, MMP-9 secretion has been shown to be triggered by PAR-2 activation (50).

Our results indicate that P1 can induce TSLP production when interacting with the nasal epithelium thus probably also during nasal vaccination. However, the pathway induced by the gp41-subunit P1 differs completely from that elicited by gp120 within the HIV-1-envelope spike during HIV-1 sexual transmission at genital sites (15, 16). Regarding its role in the nasal vaccination process, TSLP produced at the inductive site may imprint mucosal immune cells, such as DCs, so that these cells acquire an innate memory (51), prior to circulating towards the effector site at the genital mucosa, as proposed (3). Consequently, during the very first contact with HIV-1 at the genital mucosa—also the vaccine effector site—these imprinted immune cells could receive from HIV-1 envelope a signal to induce TSLP, and in turn rapidly mobilize vaccine induced memory cells to prevent infection.

On the non-lymphoid area of the mucosal surface, sub-mucosal DCs act as sentinels for monitoring ‘danger signals’ such as cytokines and chemokines induced by antigen stimulated epithelial cells. Upon activation, these DCs may penetrate epithelial tight junctions *via* trans-epithelial dendrites and uptake antigens, as characterized in the nasal epithelium (30, 52). DCs are generally considered as the major target of TSLP (53). In a previous study using TSLP as an adjuvant, nasal application at the nasal epithelial surface was sufficient to activate sub-mucosal DCs, suggesting that TSLP-induced signal is able to cross the epithelial barrier (17). Therefore, we further investigated the modulatory effect of P1 on mucosal DCs, by using a nasal ECs and DCs co-culture model to mimic the nasal mucosal environment and cell–cell contacts.

Our data reveal that P1 stimulates the expression of the co-stimulatory factors CD83 and CD86 on mucosal DCs, as well as the TSLP receptor. Considering that TSLP regulates Th2 polarization, we investigated the cytokine and chemokine profile stimulated by P1 in mucosal DCs. Our data showed that in mucosal DCs, P1 induced IL-6, IL-10, and reduced IFN-*γ*, corresponding to an anti-inflammatory Th2 response and enhancing IgA class switching. IL-8, CCL20, and CCL22 are also produced and, in turn, could induce the recruitment of immune cells for the initiation of the adaptive immune response (macrophages, lymphocytes, monocytes) to the mucosal stroma. The last factor secreted upon P1 stimulation is MMP-9 that facilitates the migration of immune cells by degrading the extracellular matrix. In addition, P1-modulated mucosal DCs lead to autologous CD4+ T cell proliferation. Thus, our results indicate that P1 activates mucosal DCs to release robust Th2 cytokines and chemokines and therefore might promote the mucosal humoral response.

TSLP produced by ECs is expected to mediate crosstalk with DCs leading to Th2 polarization of the immune response. However, we surprisingly found that ECs are not the only source of TSLP in our mucosal models. Mucosal DCs obtained in our two models, namely DC–EC and eduDC, react similarly to P1 stimulation. In particular, P1 induces a similar level of TSLP secretion and increased the expression of TSLP receptor, two elements that might form together a TSLP autocrine loop, in turn, amplifying the TSLP-induced signal. This autocrine loop is actually consistent with the clinical studies reporting that TSLP secretion and TSLP receptor expression are augmented simultaneously upon EC activation (54, 55). Such autocrine TSLP loop in DCs would sustain the source of TSLP and help to maintain the DC–epithelial crosstalk. Accordingly, we found that the ‘education’ induced by the interaction with ECs and its specific microenvironment provide unique functional features to mucosal DCs upon P1 stimulation characterized by a different immune profile compared to blood DCs. This is consistent with previous reports indicating that the immunological consequences subsequent to an adjuvant stimulation are primarily dependent on the environment in which the adjuvant is applied (41, 56). Further studies will be necessary to better understand the mechanisms involved in the epithelial ‘education’ and on P1-induced DC activation pathways.

As a final step in the characterization of P1 adjuvant function, we showed that P1 induces antigen-specific IgG and IgA at the surface of B cell in an *in vitro* immunization assay. Previous studies reported that using PBMCs depleted in immunosuppressive cells, an antigen-specific immune response can be elicited by antigen sensitization in the presence of cytokines and adjuvants (29, 31, 42, 43). Therefore, we used this *in vitro* method to evaluate the capacity of P1 to act as an adjuvant and enhance an antigen-specific humoral response using OVA as a model antigen. Our results clearly indicated that P1, when in an oligomeric state, enhanced the number of OVA-specific B cells by either favoring antigen presentation and/or T-B interaction. Both OVA-specific B-cell surface IgA and IgG were detected, whereas specific antibody secretion was undetectable at day 5/7, suggesting that in this *in vitro* model, class switching occurred, but B cells did not develop into plasmablasts. Specific antibody secretion in such *in vitro* immunization system would require additional stimulations with agents such as CD40L and other cytokines or repeated sensitization with antigen. Nevertheless, the adjuvant function of P1 should be confirmed *in vivo* upon intranasal immunization of P1-adjuvanted antigens.

**Figure 7:**
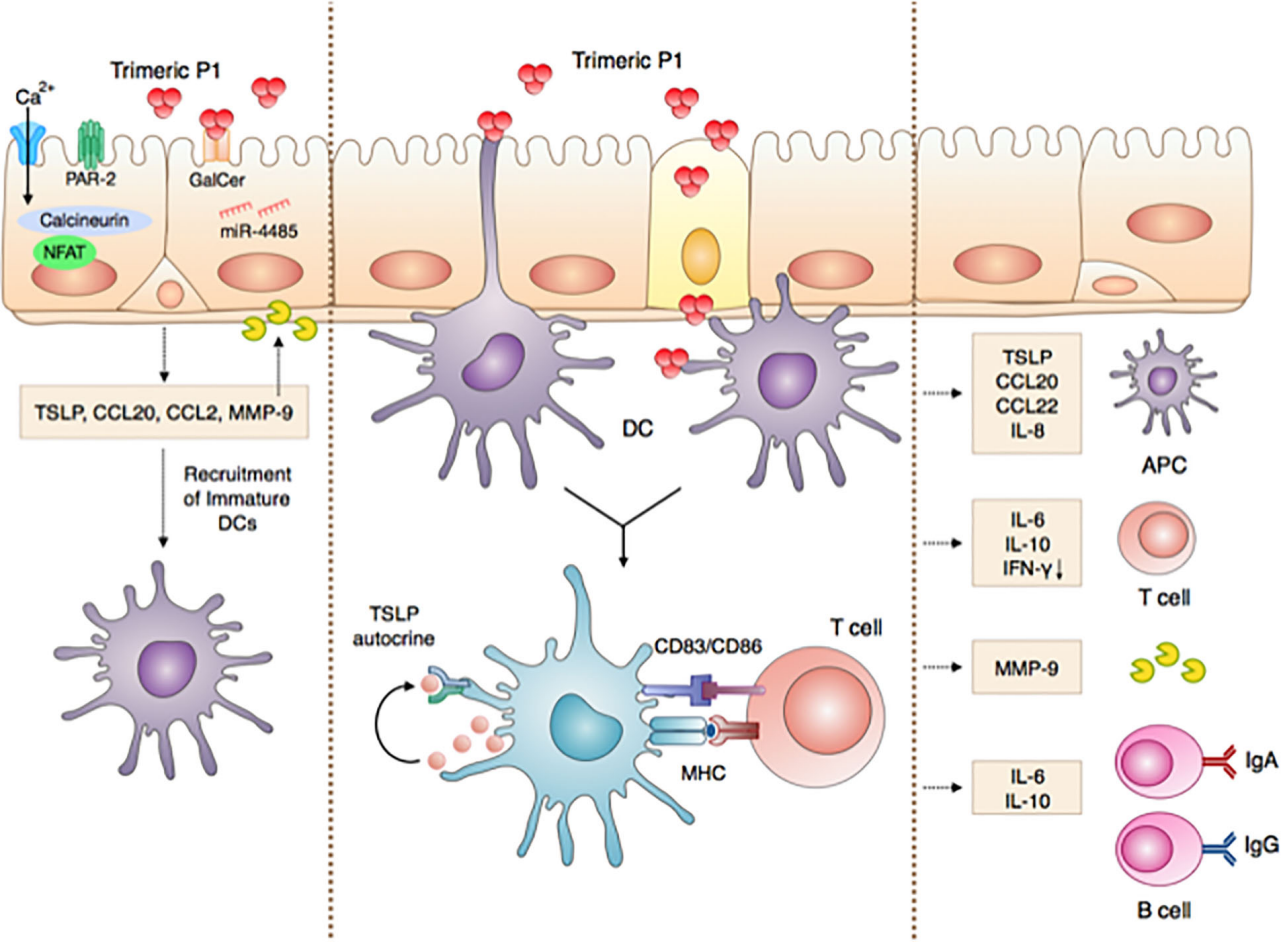
‘Chain of events’ summarizing the P1 immunomodulatory adjuvant activity in initiating innate and adaptive immune responses at the nasal mucosa. Step 1: P1 activates nasal epithelia cells to produce TSLP by interacting with GalCer, activating, in turn, miR-4485, calcium influx, PAR-2 and calcineurin, as well as the secretion of CCL20, CCL2, MMP-9, and IL-10. These cytokines and chemokines initiate the very first steps of the immune response and mediate the crosstalk between ECs and immune cells such as DCs. Step 2: P1 interacts with mucosal DCs, thereby enhancing the expression of co-stimulatory markers and TSLP receptor in an autocrine loop and favoring DC maturation. As a result, P1**-**activated mucosal DCs initiate an adaptive immune response, by eliciting chemokine release that in turn can recruit adaptive immune cells and cytokine secretions. Consequently, adaptive immunity is polarized into a Th2 and IgA preferential response. Step 3: Finally, P1-stimulated DCs promote CD4+ T cell proliferation and enhance antigen-specific B cell responses.

Altogether, our results show that in addition to its protective vaccine antigen properties contributing to full protection against repeated mucosal SHIV infection (8, 12) and to inducing in a phase I clinical trial in human mucosal P1-specific IgA with *in vitro* HIV transcytosis blocking properties (8, 12), P1 acts as an adjuvant, as we now demonstrated here. We show that P1 adjuvant activity occurs in a two-step mode of action during nasal vaccination. In the first step, P1 initiates the very first immune responses at nasal epithelial surface, by producing TSLP, a molecule considered as a strong adjuvant, together with CCL20, CCL2, MMP-9 and IL-10, cytokine determinant in the mucosal immune response. This process is initiated by P1 interaction with Galactosyl Ceramide, the epithelial receptor for HIV-1 (9–11), activates calcium influx and the PAR-2 and calcineurin pathways, and is regulated by the microRNA miR-4485. In the second step, P1 modulates mucosal DCs by inducing the expression of maturation markers, promoting chemokine release that can recruit adaptive immune cells and cytokine secretion that can polarize adaptive immunity into a Th2 and IgA preferential fashion. As a result, P1-modulated DCs promote CD4+ T cell proliferation and enhance antigen B cell-specific responses, as summarized in **Figure 7**.

Overall, the present study reveals that P1 is a multi-functional protein with a strong vaccine potential, namely as an immunogen in a fully protective HIV-1 vaccine candidate (8, 12) but also as a promising adjuvant that can combine with other mucosal vaccines.

## Data Availability Statement

The data has been uploaded to NCBI - GSE157449. Other raw data supporting the conclusions of this article will be made available by the authors, without undue reservation, to any qualified researcher.

## Author Contributions

LX, DT, and MB conceived the experiments. LX, DT, and MB performed and analyzed experiments. LX and MB wrote the manuscript. All authors contributed to the article and approved the submitted version.

## Funding

Agence Nationale de Recherche sur le SIDA et les Hepatites (AO2015-2-17046). Fondation pour la Recherche Medicale (Grant : « Equipe FRM » EQU201903007830).

## Conflict of Interest

The authors declare that the research was conducted in the absence of any commercial or financial relationships that could be construed as a potential conflict of interest.
